# Lessons from miR-143/145: the importance of cell-type localization of miRNAs

**DOI:** 10.1093/nar/gku461

**Published:** 2014-05-28

**Authors:** Oliver A. Kent, Matthew N. McCall, Toby C. Cornish, Marc K. Halushka

**Affiliations:** 1 Princess Margaret Cancer Center, University Health Network, 101 College Street, Room 8-703, Toronto Medical Discovery Tower, University of Toronto, Toronto, ON M5G 1L7, Canada; 2 University of Rochester, Department of Biostatistics, Rochester, NY 14642, USA; 3 Johns Hopkins University, Department of Pathology, Baltimore, MD 21205, USA

## Abstract

miR-143 and miR-145 are co-expressed microRNAs (miRNAs) that have been extensively studied as potential tumor suppressors. These miRNAs are highly expressed in the colon and are consistently reported as being downregulated in colorectal and other cancers. Through regulation of multiple targets, they elicit potent effects on cancer cell growth and tumorigenesis. Importantly, a recent discovery demonstrates that miR-143 and miR-145 are not expressed in colonic epithelial cells; rather, these two miRNAs are highly expressed in mesenchymal cells such as fibroblasts and smooth muscle cells. The expression patterns of miR-143 and miR-145 and other miRNAs were initially determined from tissue level data without consideration that multiple different cell types, each with their own unique miRNA expression patterns, make up each tissue. Herein, we discuss the early reports on the identification of dysregulated miR-143 and miR-145 expression in colorectal cancer and how lack of consideration of cellular composition of normal tissue led to the misconception that these miRNAs are downregulated in cancer. We evaluate mechanistic data from miR-143/145 studies in context of their cell type-restricted expression pattern and the potential of these miRNAs to be considered tumor suppressors. Further, we examine other examples of miRNAs being investigated in inappropriate cell types modulating pathways in a non-biological fashion. Our review highlights the importance of determining the cellular expression pattern of each miRNA, so that downstream studies are conducted in the appropriate cell type.

## INTRODUCTION

MicroRNAs (miRNAs) are short 18–24 nucleotide single-stranded RNAs that bind the 3′UTR of their cognate mRNA transcripts to repress or activate translation or to cause mRNA turnover and degradation (1). miRNAs have been found across all eukaryotic life forms including diverse plant and animal species where they participate in a wide range of functions including the regulation of cellular proliferation, cellular motility, differentiation and apoptosis. In humans, thousands of miRNAs have been identified and are collectively predicted to regulate at least one-third of all mRNA transcripts.

In just over a decade, our understanding of the structure and role of this important class of non-coding regulatory RNA has exploded. Investigators have characterized thousands of miRNAs, either predicting or experimentally validating thousands of their gene targets (2,3). Researchers have shown that miRNAs are organized into highly conserved families with common seed regions of about 6–8 nucleotides that determine target specificity (4). Our community now understands that each miRNA has numerous gene targets and that a given gene may be targeted by multiple miRNAs providing a combinatorial effect on regulation (5).

A great deal is also known about the genomic footprint of miRNAs. Groups have categorized miRNAs by their genomic location and have found that miRNAs are contained in both coding and non-coding genes. They can be found in introns and exons and often share their expression patterns and regulation with the host gene (6). Many miRNAs are found in polycistronic clusters that are usually under the regulation of a single promoter. A single cluster may span 100s-to-1000s of nucleotides and contain from two to dozens of miRNAs. We have also learned that secondary and tertiary structural elements of the primary transcript can influence miRNA processing and expression. For example, the miR-17–92 cluster contains six miRNAs that form a compact globular tertiary structure. The miRNAs in the cluster that are in the interior of the folded structure are processed less efficiently than miRNAs exposed on its surface (7). In addition, researchers have begun to understand the biological roles miRNAs have in regulating cellular processes—including cell signaling pathways, cell cycle control and DNA repair mechanisms—in neoplastic and non-neoplastic disease (8). Finally, studies have shown the potential for miRNA-based therapeutic agents in the treatment of human disease (9,10).

Despite this decade of advancement, what has not been studied in any comprehensive way is what cell types actually express specific miRNAs. It turns out that this matters a great deal. As we demonstrate, having knowledge of cell type-restricted miRNA expression would allow miRNA functional studies to be performed in appropriate cell types so the findings from these experiments would carry biological relevance. While this is an endemic problem in the miRNA community, it is also known in other RNA communities, where attempts at tissue deconvolution or understanding single-cell mRNA expression patterns have been reported and may represent a new way forward in miRNA studies (11–13).

### miR-143/145 are not highly expressed in non-neoplastic epithelial cells

**Figure 1.**
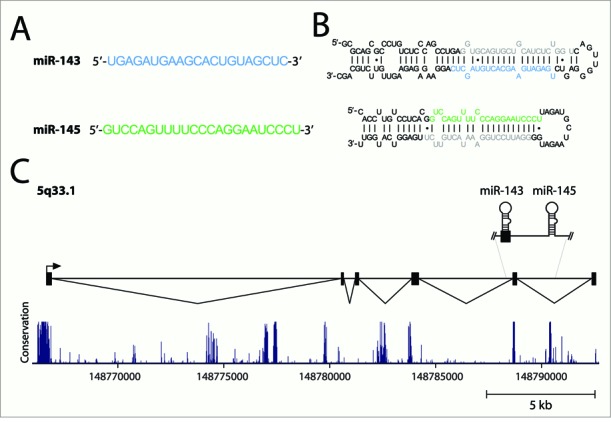
The miR-143/145 cluster. (**A**) Mature miRNA sequences of miR-143 and miR-145. (**B**) The proposed secondary structures of the pre-miR-143 and pre-miR-145 stem loops. The mature miRNA sequences are blue (miR-143) and green (miR-145). miRNA-star sequences are shown in gray. (**C**) The genomic organization and major primary transcript structure of the miR-143/145 cluster. The minor transcript (not shown) lacks exon 2. The plot depicted below the transcript shows evolutionary conservation (UCSC Genome Browser 28 species conservation track, NCBI36/hg18 assembly).

The expression of the miR-143/145 cluster represents a clear example of how understanding cell-type expression patterns of miRNAs is of vital importance. miRNAs miR-143 and miR-145 form a bicistronic cluster in 5q33.1 (Figure 1). These two miRNAs have been deemed ‘tumor suppressors’ and have been studied extensively for their role in neoplastic pathways in epithelial cell malignancies (14–16). miR-143, in particular, is one of the most abundant miRNAs in colonic tissue. Its ‘loss’ from a normal epithelial cell during transformation to the malignant state is commonly accepted.

Chivukula *et al.* have demonstrated that miR-143/145 are not expressed in intestinal epithelial cells (17). In this study, the authors generated constitutional or tissue-restricted mice with a deletion of miR-143/145. They reported no developmental phenotype but showed that absence of these miRNAs led to a lethal failure of intestinal regeneration after administration of dextran sulfate sodium which induces a normally survivable injury-regeneration sequence. *In situ* hybridization studies demonstrated miR-143/145 expression exclusively in mesenchymal cells. Quantitative RT-PCR confirmed this with nearly undetectable levels in epithelial preparations. Using a series of elegant mouse models, this group further demonstrated that the role of miR-143/145 in intestinal wound repair was exclusively through their functions in the mesenchymal cell component. This work advances the discovery of a major role of miR-143/145 in injury response. Previously, Xin *et al.* knocked out miR-143/145 in mice which abrogated medial remodeling in a carotid artery ligation vascular tissue injury model (18). A separate balloon-injury model of the carotid artery demonstrated the opposite effect where less neointimal formation was observed when adenovirus-delivered miR-143 and miR-145 was present compared to a wild-type mouse (19).

The Chivukula *et al.* discovery is certain to be controversial as other groups have reported miR-143 expression in epithelial cells including observing the presence of miR-143 expression by LNA-ISH in maturing murine colonic epithelium (20). We evaluated this independently, performing small RNA RNA-seq, with library preparation using the Illumina TruSeq Small RNA Sample Preparation kit and sequencing libraries in a multiplex fashion on a HiSeq2000. We analyzed the sequence read data using a modification of miRDeep2 (21). We determined miR-143/145 levels obtained from flow-sorted EPCAM^+^ epithelial cells, isolated red blood cells and cell-cultured endothelial, fibroblast and smooth muscle cells (SMCs) (22). We found miR-143-3p to be among the most abundant miRNAs in fibroblasts and SMCs (Table 1). We also found significantly lower levels of miR-143-3p (33 or greater fold lower than fibroblasts and SMCs) in other cell types including epithelial cells, endothelial cells and red blood cells. We cannot fully explain these low levels, but hypothesize it may represent mesenchymal contamination or a leaky promoter. miR-145 had low levels of expression in both mesenchymal cell types and was negligible in other cell types. In light of these findings, we must revisit the early miR-143/145 publications to understand why a reduction in miR-143/145 signal in colon cancer tissue was attributed to a reduction in epithelial cell expression.

**Table 1.** Expression profiles of miRNAs across different cell types

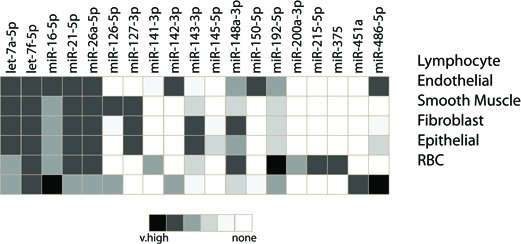																			

### A brief history of miR-143/145 colonic expression

The initial discovery of miR-143 and miR-145 came from Michael *et al.* in the seminal study that both identified their transcripts in colonic adenocarcinoma tissue and demonstrated a reduced level compared to normal colonic mucosa (23). The authors purified total RNA from both colonic adenocarcinomas and a matching normal colonic mucosa taken at the time of tumor resection. Small RNAs were then fractionated, cloned and sequenced. Several of the resulting clones were homologous to known murine miRNA sequences, miR-143 and miR-145, and these were found at lower levels in the neoplastic specimens compared to the matched normals.

The finding of differential miR-143 and miR-145 expression between normal colon and colorectal cancer biopsies was confirmed by the study of Akao *et al.* (14). Similar to the previous study, Akao *et al.* performed northern blot analysis of miR-143 and miR-145 expression in total RNA isolated from tumors and adjacent normal tissue. The authors also demonstrated that neither miR-143 nor miR-145 were expressed in a panel of epithelium-derived cancer cell lines, whereas both miRNAs were readily detectible in normal human tissue. Believing their data indicated these miRNAs were reduced in malignant epithelial cells, they introduced both miR-143 and miR-145 into the colorectal cancer cell lines DLD-1 and SW480 which had the effect of significantly decreasing cellular proliferation. They then confirmed that the previously predicted miR-143 target *ERK-5* was translationally repressed by exogenously transfected miR-143 precursor in these cell lines. The authors interpreted these results as suggesting that miR-143/145 expression decreases as epithelial cells differentiate into a malignant phenotype.

This general theme of comparing either tumor tissue/cell lines to normal tissue was performed for a variety of other malignancies. Lui *et al.* demonstrated reduced expression of miR-143 in six cervical cancer cell lines compared to five samples of normal cervical tissue (24). Chen *et al.* reported reduced miR-143 and miR-145 in 13 nasopharyngeal carcinomas compared to nine adjacent samples of normal nasopharynx tissue (25). Takagi *et al.* reported reduced miR-143/145 in a comparison of 43 matched tumor/adjacent normal gastric carcinoma cases (16). Szczyrba *et al.* demonstrated ∼4-fold downregulation of miR-143/145 in pools of dissected prostate cancer tissues containing >70% tumor cells versus matched normal prostate tissues (26). Thus, a general sense of miR-143/145 loss in epithelial neoplasms was established. However, a greater appreciation of the cellular makeup of the colon or other tissues may have led to a different interpretation in these papers.

### The cellular composition of colon

**Figure 2.**
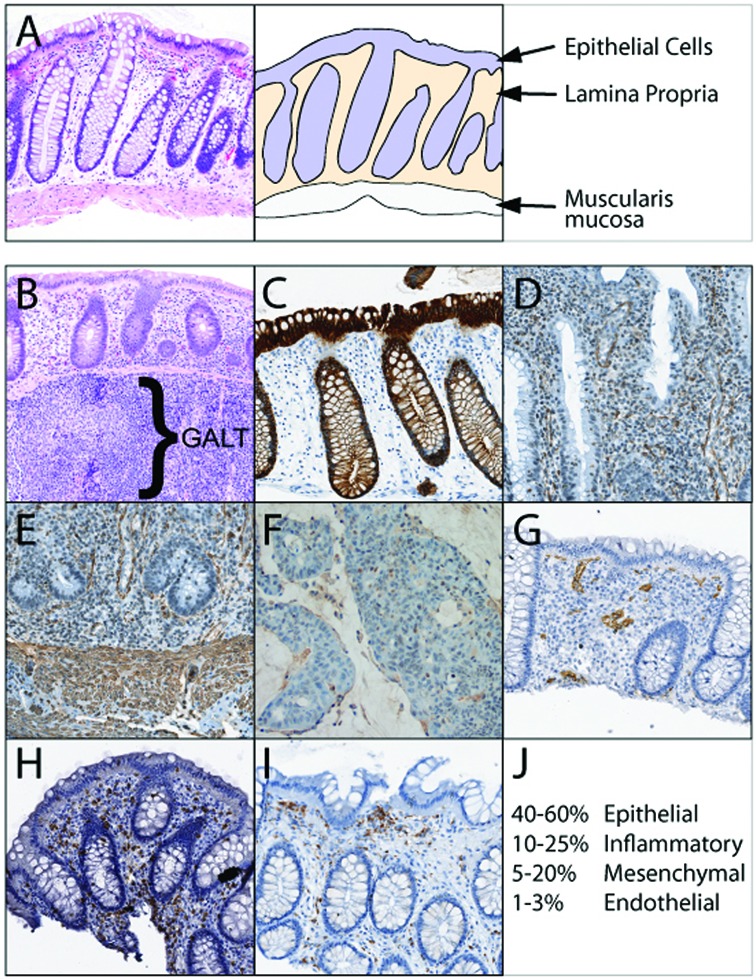
Cellular components of the colonic mucosa with direct visualization of unique cell types by immunohistochemistry. (**A**) Representative hematoxylin & eosin image of a typical normal colonic biopsy (left) and a schematic representation of the colon biopsy (right) indicating cell types found in the tissue. (**B**) Normal gut-associated lymphoid tissue (GALT) containing collections of lymphocytes can be found in the mucosa and superficial submucosa of some biopsies. (**C**) AE1/AE3 staining of the epithelial component of the colonic mucosa. (**D**) Smooth muscle actin (SMA) staining of normal colon demonstrating SMC, pericyte and fibroblast staining within the lamina propria and (**E**) more extensive SMA staining of SMCs in the muscularis mucosa. (**F**) SMA staining in an exophytic colon adenocarcinoma is greatly reduced compared to normal tissue. (**G**) CD34 staining demonstrating endothelial cells within the lamina propria. (**H**) CD3 staining demonstrating T lymphocytes scattered within the lamina propria. (**I**) CD68 staining demonstrating predominantly macrophages underlying the surface epithelium. (**J**) Image analysis of these staining patterns across multiple routine colonic biopsies identified typical ratios of cellular composition of colon. These ratios can be markedly different depending on the amount of muscularis mucosa and GALT present in the biopsy.

A biopsy or superficial resection of colon is usually limited to the mucosa but may also include a small amount of superficial submucosa. In the normal colon, these biopsies are composed principally of epithelium and lamina propria. They usually contain some amount of muscularis mucosa (the deepest portion of the mucosa) and may even contain gut-associated lymphoid tissue (GALT) (Figure 2A and B). The colonic epithelium is comprised of absorptive and goblet-type epithelial cells. The lamina propria contains multiple cell types including mixed inflammatory cells, endothelial cells, fibroblasts, SMCs and pericytes. The muscularis mucosa contains primarily SMCs (Figure 2C). Even the earliest of adenocarcinomas obliterates the normal histology of the colon. A sample from an exophytic adenocarcinoma would contain primarily malignant epithelial cells, rarer fibroblasts and inflammatory cells (depending on the specific type of tumor) but no SMCs.

For the purposes of demonstration, we stained a colonic mucosal biopsy, using standard immunohistochemistry using antibodies to proteins that mark specific cell types. We stained colonic tissue for smooth muscle actin (SMA), a marker of mesenchymal cells (Figure 2D and E). A significant number of cells stained positive. In contrast, the exophytic adenocarcinoma biopsy has minimal staining for SMA (Figure 2F). CD34, CD3 and CD68 stain endothelial cells, T cells and macrophages respectively (Figure 2G–I).

As miR-143 and miR-145 are expressed at high levels in SMCs and fibroblasts (i.e. SMA^+^ cells) these components of the biopsy would be expected to contribute highly to the expression of miR-143 and miR-145 in normal colon biopsies (17). The reduction or lack of these same cell types in adenocarcinoma (Figure 2F) would result in lower miR-143 and miR-145 levels. In other words, the large shift in cellular composition in adenocarcinoma adequately explains the observed reduction in miR-143 and miR-145 when adenocarcinoma is compared to normal colon. One would expect this result based purely on the changing ratio of cell types present in the tissue. This variation in the presence of mesenchymal cells is also essentially true for all non-desmoplastic carcinomas described above.

### What miR-143/145 data is biologically relevant?

This reinterpretation of the source of miR-143/145 in tumor and normal tissues forces us to reconsider miR-143/145 functional studies. Following the initial publications, many studies were undertaken to explain the activity of miR-143/145 in epithelial cell lines derived from colon and other epithelial cancers. As the new evidence indicates that miR-143/145 are principally expressed in mesenchymal cells with no evidence of intestinal epithelial cell functionality, epithelial cell lines are not ideal cell types to assay function of these miRNAs. Then what is to be done with all of the data generated in epithelial malignancies?

For direct gene-miRNA interactions, we generally believe that any gene known to be expressed in mesenchymal cells and shown to be modulated by miR-143/145 in any cellular system is biologically valid. Those described specifically in mesenchymal cell studies (SMC, fibroblast, adipocyte, myofibroblast, etc.) are definitely biologically relevant. For targets identified from epithelial cell studies, a simple bioinformatics or publication search will identify which mRNA targets are also expressed in mesenchymal cells (Table 2)(27,28). Those mRNAs that are not expressed in mesenchymal cells but shown to be modulated by miR-143/145 (ex. *MUC1*) are unlikely to be biological targets (29). The biological relevance of directed miR-143/145 expression on the modulation of malignant phenotypes (such as invasion, migration and proliferation) in epithelial malignancies is particularly difficult to determine. However, these described activities are potentially important as therapeutic targets, as we describe below.

**Table 2.** Examples of known gene targets of miR-143 and miR-145

Gene	Biologic system of discovery	Gene expression in mesenchymal cells	Targeted by	Reference
*ELK-1*	Fibroblast/VSMC	Yes	miR-143	(30)
*ERK-5*	Adipocytes	Yes	miR-145	(31)
*FLI*	Colon cancer	Yes	miR-145	(27)
*IGFBP5*	Myofibroblasts	Yes	miR-143	(17)
*K-RAS*	Epithelial malignancy	Yes	miR-143	(32)
*MUC1*	Epithelial malignancy	No	miR-145	(29)
*MYOCD*	Fibroblast/VSMC	Yes	miR-145	(30)
*RREB-1*	Epithelial malignancy	Yes	miR-145	(33)
*VCAN*	Muscle	Yes	miR-143	(28)

### The role of miR-143/145 in mesenchymal cells

In addition to the new role of miR-143/145 in intestinal wound repair described above (17), other activities of these miRNAs have been reported in mesenchymal cells. Both miRNAs have an established function in regulating differentiation of multipotent and pluripotent stem cells. A critical role for miR-143/145 has been identified in vascular SMCs. Through downregulation of Kruppel-like factor 4 (Klf4), myocardin, Elk-1 and numerous other targets, miR-143/145 can direct the reprogramming of fibroblasts into SMCs during development (30,34). In addition, miR-145 acts to silence multiple pluripotency factors during the switch from stem cell self-renewal to lineage commitment (35). miR-145 was found to be highly expressed in embryoid bodies, an aggregate of pluripotent stem cells. Through the downregulation of the pluripotency factors OCT4, SOX2, and Klf4, miR-145 inhibits embryonic stem cell self-renewal and induces lineage-restricted differentiation. miR-143 has also been shown to play a role in cellular differentiation. Using a miRNA microarray, miR-143 was identified to exhibit increased expression in maturing adipocytes and inhibition of miR-143 effectively attenuated differentiation (31). The mechanism may involve downregulation of miR-143 targets *ERK-5*, identified in cultured human pre-adipocytes, and fibroblast growth factor 7 (FGF7) in murine adipogenesis (31,36).

### The miR-143/K-RAS story

**Figure 3.**
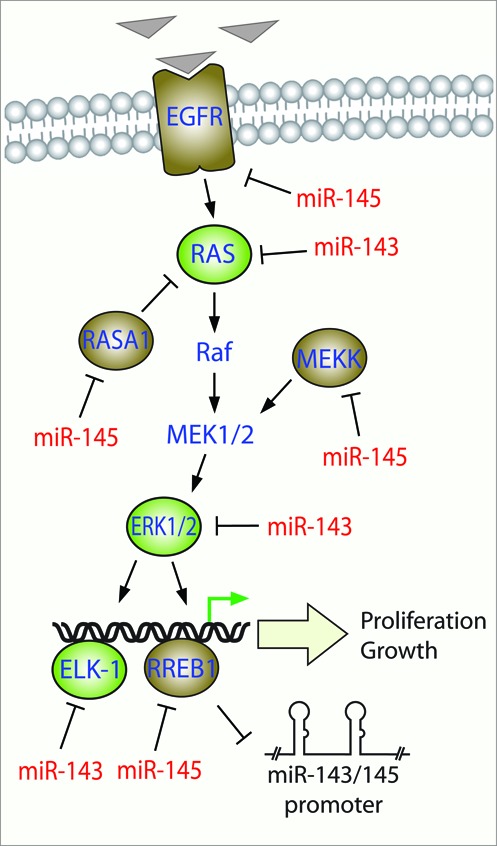
miR-143/145 modulate signaling through the Ras-MAPK pathway. Extracellular mitogenic signals (triangles) leads to activation of Ras and the signaling through the Raf-MEK-ERK (MAPK) cascade to activate transcription factors such as Elk-1 and RREB1 resulting in proliferation and growth responses. Targets of miR-143 and miR-145 are shown in green and brown respectively. The miR-143/145 proximal promoter is negatively regulated by K-Ras-RREB1 feedback loop.

Arguably, one of the most important examples of miR-143/145 regulation is the modulation of *K-RAS* signaling (Figure 3). K-Ras is a small GTPase that regulates multiple cellular signaling pathways, has constitutively active mutant forms in pancreatic and colorectal cancers, and is fundamental to epithelial transformation in cancer (37). Importantly, *K-RAS* was validated to be a miR-143 target in the colorectal cancer cell line Lovo by treatment with a miR-143 mimic (32). Although the regulation of *K-RAS* by miR-143 was identified in an epithelial cell type, miR-143 may also regulate *K-RAS* in mesenchymal cells, which have been found to express functionally active Ras. For example, it has been shown that Ras guanine exchange factors Sos1 and its adaptor Grb2 couple fibroblast growth factor (FGF) signaling to activation of the Ras-MAPK pathway during mammalian development and drive embryonic stem cell lineage commitment (38). The mouse fibroblast cell line NIH-3T3, can be transformed with oncogenic *K-RAS.* We have shown that these cells lose the expression of miR-143/145 via negative promoter regulation involving the Ras responsive element binding protein (RREB-1) downstream of K-Ras (33). Furthermore, miR-143 and miR-145 have been shown to be downregulated in well-differentiated and dedifferentiated liposarcomas, as compared to normal fat tissue (all cells of mesenchymal origin). miR-143 and miR-145 exhibit tumor suppressor activity when re-expressed in a liposarcoma cell line (39). Although dysregulation of *RAS* genes by induced miR-143 expression was not identified in that study, activating *K-RAS* mutations have been found in liposarcoma and other sarcomas (40). This suggests that *K-RAS*, although identified as a target of miR-143 in a biologically irrelevant context, is still a likely target with biological relevance.

### A role for therapeutic miR-143/145 in epithelial malignancies

Numerous studies have explored the role of enforced miR-143/145 expression in epithelial malignancies. Ectopic miR-143/145 expression can potently deregulate signaling through the Ras-Raf-MEK-ERK (MAPK cascade) in addition to the modulation of other proliferative signaling networks such as the PI3K-AKT pathway, TGF-β signaling via targeting TGF-β activated kinase (TAK1, MAP3K7) and Jun-N-terminal Kinase (JNK) (41,42). Induced miR-143/145 expression in various epithelial cancers such as cervical, colon, gastric, pancreatic carcinomas and adenocarcinomas block tumorigenesis both *in vitro* and *in vivo* (16,43–45). Enforced expression of miR-143/145 via systemic intravenous delivery with nanoparticles has been shown to inhibit tumor growth of orthotopic pancreatic xenografts by downregulation of miR-143 and miR-145 targets *K-RAS* and *RREB-1*, respectively (10). Therefore, the tumor suppressive nature of these two miRNAs cannot be ignored, and these studies collectively highlight the potential to use miR-143/145 to target genes or networks commonly dysregulated in epithelial cancers.

### Inappropriate directed cell-type expression is not exclusive to miR-143/145

The enforced expression of miRNAs in inappropriate cell types are a result of studies that fail to take into account the cellular composition of tissues. Simply put: miRNAs are expressed in cells; they are not expressed in tissues. All tissues contain multiple cell types, as discussed above for the example of normal colon (See Figure 2). These include cell types resident in the organ (ex. epithelial cells, mesenchymal cells, endothelial cells, pericytes), those ‘just passing through’ (ex. erythrocytes, neutrophils, monocytes) and those that migrate into or proliferate in the organ due to disease (ex. fibroblasts, lymphocytes, plasma cells, macrophages). A homogenized tissue will contain all of these cell types in ratios that can vary widely by individual, disease process, or location within an organ. Certain miRNAs, such as miR-21 and let-7f, have universally high cellular expression. Therefore, the tissue-level signal obtained from these miRNAs is a composite of all cells within the organ and less a function of the ratio of each cell type. Other miRNAs, such as miR-143, miR-145, miR-150 and miR-451a, are restricted to expression in a single (or small number of) cell type(s) (Table 1). The tissue-level signal is therefore a function of both the cell type's abundance in the tissue and the miRNA expression level in the cell type. Thus, understanding the cellular pattern of miRNA expression is critical to interpreting tissue level expression data.

Projects that have attempted to catalog cellular miRNA expression data have generally done so in tumor cell lines or other immortalized cell lines (46,47). It is unlikely these genetically aberrant cell types faithfully recapitulate the miRNA expression of a normal cell. For the purpose of this review, we investigated the miRNA expression profiles of all Gene Expression Omnibus (GEO) available Agilent miRNA arrays obtained from cancer cell lines with methods described previously (48). We provide data from 29 cell lines covering seven unique epithelial neoplasms (Table 3). Where we found multiple cell lines for the same malignancy, we could demonstrate clear heterogeneity in expression (ex. miR-200a and miR-141-3p). We also observed cell line changes in expected miRNA expression such as the loss of expression of the prototypically cell-specific and highly expressed miRNA, miR-122, in six of seven hepatocellular carcinoma cell lines (49). We also noted the presence of some miRNA expression in cancer cell lines that are not reported in corresponding normal epithelial cells (ex. miR-126-5p and miR-142-3p). We use this data to highlight potential concerns of using cancer cell lines as a surrogate of primary cell types.

**Table 3.** Expression profiles of miRNAs across different cancer cell lines

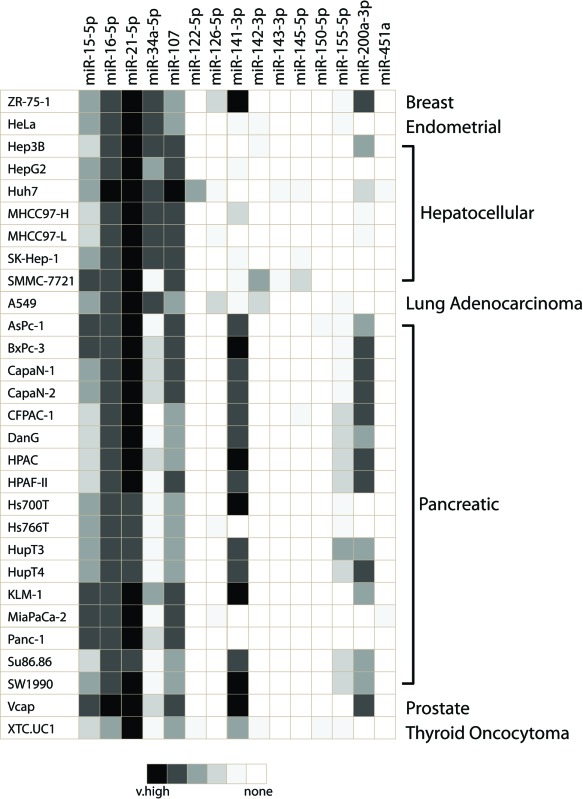																

We believe from our work and the work of others that miR-451a is expressed exclusively in erythrocytes (red blood cells), miR-150 in leukocytes, and miR-126 in endothelial cells and perhaps moderately in some leukocytes (50,51). However, as we discuss below, tissue level data have implicated these miRNAs as being altered in disease, causing them to be investigated in inappropriate cell types. We provide examples of three miRNAs—miR-451a, miR-150 and miR-126—that have been studied in this fashion.

### miR-451a—an erythrocyte specific microRNA

The first study reporting miR-451a was in 2005, where it was found to be in a bicistronic cluster with miR-144 (50). A year later, it was described as being the ‘principal RNA found in erythrocytes’ due to its ability to be detected from as little as 1 ng of total RNA. The same authors excluded miR-451a from a series of immortalized cell lines that represented progenitor stages of blood cell maturation (52). Our two comparisons of primary cell types demonstrate miR-451a is expressed exclusively in red blood cells [Table 1 and (48)].

Erythrocytes, red blood cells, are ubiquitous in every human tissue since they are constantly delivering oxygen to the tissue. Thus, all human tissue samples will be contaminated with erythrocytes and, by logical extension, miR-451a. Therefore, it is not surprising that miR-451a is frequently identified as altered in tissue comparisons and even in serum/plasma biomarker studies of neoplastic and non-neoplastic disease (48,53). While these alterations in miR-451a expression at the tissue level should be understood as changes in trapped red blood cells, they are frequently the focus of studies that erroneously interpret that data as altered miR-451a expression in epithelial cells. For example, miR-451a has been shown to be decreased in esophageal cancer tissue and several other epithelial cancers. Analogous to the miR-143/145 story, the investigators found that upregulated expression of miR-451a induced apoptosis and suppressed cell proliferation, invasion and metastasis in the esophageal carcinoma cell line EC9706 and modulated miR-451a targets Bcl-2, AKT and p-AKT (54). In another study, miR-451a levels correlated with colon cancer disease free survival and the authors expressed miR-451a in a colorectal adenocarcinoma cell line (DLD1), where it reduced metabolic activity and sensitized the cells to radiation therapy (55). Expression of the miR-144/451a cluster was also reduced in liposarcomas compared to normal fat and enforced miR-451a expression in liposarcomas had tumor suppressor effects (56).

There have been at least two studies that report miR-451a expression in cultured neoplastic cells. In one study miR-451a was expressed 2.2-fold higher in an ovarian drug resistant cancer cell line (A2780DX5), as compared to the non-drug resistant cell line (A2780) (57). Unfortunately, this array data is not in the public domain and it is not clear if this was a robustly expressed miRNA or an example of finding a spurious signal in the noise. A second study claimed an increase in miR-451a expression in the colon carcinoma cell line (T84) during epithelial cell polarity formation. At this time, there is no compelling evidence that miR-451a is expressed outside of erythrocytes (Tables 1 and 3). Thus, studies of miR-451a in epithelial cell pathways are biologically questionable. Additionally, miR-451a is unlikely to serve as a useful biomarker of cancer or non-neoplastic disease unless it is used as a surrogate of hematocrit levels (48,53,58).

### miR-150—a leukocyte microRNA

miR-150 was described in 2007 as being a B lymphocyte and T lymphocyte miRNA that controls B cell differentiation (59,60). This miRNA is highly expressed in leukocytes, but is not found in other cell types (Table 1). Unfortunately, its specific expression pattern is underappreciated and therefore had been misinterpreted in disease states that incite inflammation.

In a study of myocardial infarction, miR-150 expression was reported to be increased in the plasma of patients exhibiting cardiac remodeling (61). This led the authors to investigate miR-150 expression levels in a myocardial infarction animal model, where it was increased in the infarct area. The authors concluded ‘miR-150 is expressed in the heart and is upregulated in infarcted tissues’. Here, the authors failed to take into account the well-known migration of inflammatory cells into the infarct zone as an explanation of the miR-150 increase.

In a mouse model of induced colitis, miR-150 expression was elevated, a finding that was confirmed in human ulcerative colitis samples (62). Again, the authors did not appreciate that the influx of inflammatory cells in these inflammatory conditions could account for the increased miR-150 levels. As a result, the authors expressed miR-150 in the colorectal adenocarcinoma cell line (HT29) where miR-150 was reported to downregulate *c-MYB* and *BCL-2*.

A separate group explored miR-150 in colon cancer (63). They described ‘downregulation’ of miR-150 in both primary and metastatic colon cancer tissues when compared to normal colonic mucosa—a tissue known to be rich in inflammatory cells. They further reported that miR-150 was involved with miR-182 and miR-183 in reprogramming energy metabolism. In our analysis of cancer cell lines, miR-150 was noted to have very low expression in a thyroid oncocytoma cell line but was otherwise absent in malignant epithelial cell lines (Table 3).

### miR-126—an endothelial miRNA

miR-126 is an intronic miRNA with well-described functions in endothelial cells (64). It is among the most highly expressed miRNAs in endothelial cells—a cell-type abundant in nearly all tissues (51,65-66). In addition, miR-126 may also be expressed at low levels in leukocytes (51), but is not expressed in non-endothelial epithelial cells or mesenchymal cells (Table 1). Contrary to the non-neoplastic data, miR-126 has been reported as present in malignant epithelial cell lines, such as breast tumor lines (Table 3). Curiously, studies of breast cancer cell lines MCF10A and MDA-MB-231 both report that miR-126 expression is lost as the cells take on a more malignant phenotype (67,68). In their separate mouse models, restoring miR-126 in MDA-MB-231 or the murine mammary tumor cell line 4T1, decreased lung metastases.

In other cancer studies, miR-126 expression was inferred to be altered in disease states based on tumor/normal tissue comparisons, leading to further studies investigating the effects of enforced expression of miR-126 in cell types including HT-29 cells, Panc-1 and AsPC-1 epithelial pancreatic cancer cells, and SGC-7901 gastric cancer cells (69–72). From these different experiments, miR-126 was shown to inhibit RhoA/ROCK signaling, lower NF-κB inhibitor IκBα levels, reduce ADAM9, target Crk and suppress proliferation and invasion of these cancer cell lines. As seen with miR-451a and miR-150, it is difficult to know if these are biologically relevant activities of miR-126.

## CONCLUSION

The miR-143/145 story highlights the importance of establishing the presence of miRNA expression in each cell-type within the system of study. Understanding miRNA expression at the cellular level has lagged considerably behind other important miRNA discoveries. Large scale miRNA expression profiling and discovery studies have been performed predominantly in tissues or neoplastic cell lines (46,47). Even the landmark study of Lu *et al.* demonstrating the power of miRNAs to classify tumor types, unknowingly utilized cell-specific miRNAs found in supporting or stromal cells to help segment their cancer data (73). Due to inherent cellular heterogeneity, tissue data fails to identify the specific cellular origins of miRNA expression. Neoplastic or otherwise immortalized cell lines are also less than ideal, as they have known major pathway abnormalities that result in clearly altered gene expression profiles. We show that miRNA expression levels can vary widely between normal and malignant cell lines derived from the same organ (Tables 1 and 3).

The thorough characterization of miR-143/145 expression by Chivukula *et al.* and our confirmation of those findings by RNA-seq of flow-sorted EPCAM^+^ colonic epithelial cells demonstrate that mesenchymal cells are responsible for the colonic expression of miR-143/145 (17). Yet, numerous miRNA functional studies have been based upon the hypothesis that miR-143/145 was lost from normal epithelial cells as they acquired malignant features and that restoring miR-143/145 would restore normal cellular activity. Indeed, these studies have shown a variety of positive functional effects of miR-143/145 on tumor cells. We believe this demonstrates that a miRNA overexpressed in a non-native cell-type will find a gene to regulate and will alter a function of the cell. Inappropriate enforced expression of miRNAs is an impediment to understanding the true biological function of miRNAs. The five miRNAs we highlight are a small sampling of a larger problem.

In the future, scientists need to be cognizant of the cellular composition of tissues and apply this information to the study of miRNA expression. miRNA expression misinformation is complicating our knowledge of miRNA function and biology. Studies where tissue is collected and homogenized for RNA or protein expression analysis need to be evaluated for alterations in cellular composition. For many diseases, there is an increase in inflammatory cells, fibrosis and altered ratios of epithelial cells. Elucidating these changes may require including pathologists or others trained in histology on the research team. Recognition of major cell ratio differences between disease and non-diseased tissues challenges a fundamental belief held by many that miRNA modulation in disease is the result of cellular changes of miRNA expression in the intrinsic cells of the tissue. As we highlight, altered cellular composition is also a major factor.

In summation, we use the recent findings of Chivukula *et al.* that the colonic miR-143/145 expression signal is the result of mesenchymal cell expression to revisit our understanding of these important miRNAs and highlight our need to understand miRNA expression at the cellular level. Before we move into new and exciting areas of miRNA discovery, it will be critically important to determine the cellular source of each miRNA signal so that biologically appropriate connections between miRNA and target genes can be established.
